# Gender-specific assessment of lipid profiles correlation with serum uric acid in non-dialysis chronic kidney disease patients: prospective observational cross-sectional study

**DOI:** 10.3389/fendo.2025.1641978

**Published:** 2025-07-30

**Authors:** Yousuf Abdulkarim Waheed, Huanhuan Yin, Jie Liu, Shifaa Almayahe, Maryam Bishdary, Karthick Kumaran Munisamy Selvam, Syed Muhammad Farrukh, Shulin Li, Yanping Wang, Disheng Wang, Xinglei Zhou, Dong Sun

**Affiliations:** 1 Department of Nephrology, Affiliated Hospital of Xuzhou Medical University, Xuzhou, China; 2 Clinical Research Center for Kidney Disease of Xuzhou Medical University, Xuzhou, China; 3 Department of Nephrology, Fengxian People’s Hospital, Xuzhou, China; 4 Department of Nephrology, the Second Affiliated Hospital of Xuzhou Medical University, Xuzhou, China; 5 Medical College, University of Fallujah, AL Anbar, Iraq; 6 Central Michigan University College of Medicine, Mount Pleasant, MI, United States; 7 Medical College, Xuzhou Medical University, Xuzhou, China; 8 Department of Internal Medicine and Diagnostics, Xuzhou Medical University, Xuzhou, China

**Keywords:** serum uric acid, chronic kidney disease, dyslipidemia, hyperuricemia, cardiovascular risk

## Abstract

**Background:**

Serum uric acid (SUA) serves as an important marker for assessing kidney function in chronic kidney disease (CKD) patients. Emerging reports suggest a potential relationship between SUA and dyslipidemia. The study aims to examine the correlation between SUA and lipid profiles in CKD population.

**Method:**

We conducted a multicenter, prospective observational cross-sectional study, enrolled n=374 stages 1/4 CKD participants were stratified by gender into (n=210 males and n=164 females). Using a multistage stratified sampling method based on age and SUA to examine the differences among groups, Spearman’s correlation and linear regression analysis were utilized to study the association between SUA and lipid profiles, and multivariate analysis to determine the effect of SUA quartiles on multiple dependent variables collectively.

**Results:**

LDL-c was positively correlated with SUA levels, with Spearman’s correlation coefficients of R=0.3553 in males and 0.5137 in females (both with *P<0.0001).* TG also showed a positive correlation, with coefficients R=0.1797 in males and 0.5115 in females (*P<0.0091* and *P<0.0001*, respectively). Similarly, TC showed a positive correlation R=0.2979 in males and 0.2741 in females (*P<0.0001* and *P<0.0004*, respectively), while HDL-c showed an inverse correlation with SUA correlation coefficients R=-0.3445 in males and -0.4055 in females (both with *P<0.0001*). The age-stratified analysis indicated that LDL-c was higher in individuals aged ≥70 compared to younger groups (*P<0.002*), while HDL-c was higher in the 20–29 age group; with (*P<0.031*). *Post-hoc* univariate tests revealed significant between quartile differences for all lipid variables (all *P ≤ 0.05*).

**Conclusion:**

In CKD population, SUA levels were positively associated with LDL-c, TC, and TG, while inversely associated with HDL-c. Additionally, lipid profiles were identified as significant predictors of SUA levels. Furthermore, the graded associations of SUA quartiles with lipid profiles suggest that SUA may be employed as a biomarker of dyslipidemia risk among this population.

## Graphical Abstract

**Figure:**
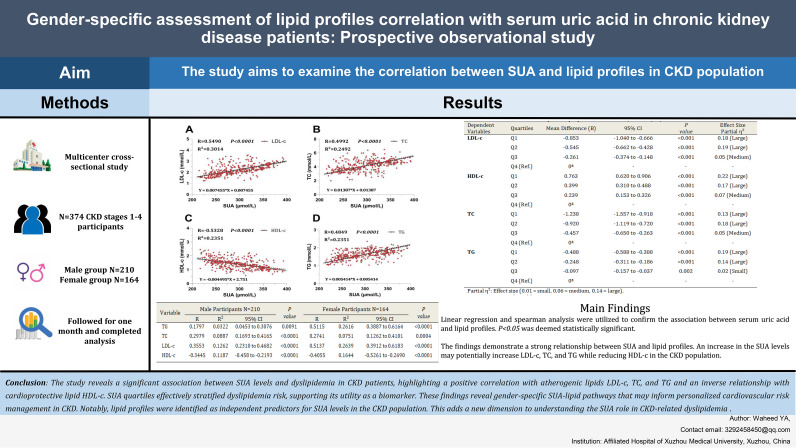

## Introduction

The end-product of purine metabolism is serum uric acid (SUA), produced in the liver and ultimately excreted via the kidneys throughout the body (1–3). Mostly, it is formed by endogenous synthesis with less being sourced externally (4). Abnormalities in either excessive production or under-excretion define the causes behind hyperuricemia (HUA). The definition for HUA diagnosis in China states the cut-off value for SUA concentration >420 μmol/L, specifically applied to male and female patients (5). According to one meta-analysis, the estimated aggregated prevalence of HUA in mainland China was found to be 13.3% (95% CI: 11.9-16.4%) (6).

SUA’s association with kidney stones is well established and significantly correlates with chronic kidney disease (CKD) (7). HUA is frequent in patients with CKD and constitutes an increase in SUA level as a result of a decrease in the estimated glomerular filtration rate (eGFR). The considered basis for such an association may be the crystallization of urate within renal tubules or in the extrarenal system, with a special emphasis on obese individuals (8). Recent studies have further supported the relationship between elevated SUA levels and initiation and progression of CKD, as well as increased cardiovascular diseases (CVD), hypertension (HTN), diabetes mellitus, metabolic syndrome, and cognitive decline (9, 10). A meta-analysis showed that the increased SUA concentrations were significantly linked with the development of metabolic syndromes, regardless of individual study characteristics (11).

Several studies have investigated this phenomenon, yielding significant insights into the dynamic relationships between SUA and lipid profiles. HUA is commonly recognized as a key risk factor for dyslipidemia. Various studies have shown a positive correlation between SUA levels and lipid profiles in adult populations (12). For instance, a study conducted in Bangladesh revealed a significant positive relationship between SUA levels and lipid profiles among adults (13). Similarly, research by Peng et al. (14) in the US found a strong correlation between lipid profiles and SUA levels in the adult population. In addition, high levels of SUA were also considered a risk factor for hypertriglyceridemia (15). However, a recent study on 409 obese Chinese with BMI of >24 kg/m2 conducted to explore this relationship revealed that SUA has no close association with metabolic diseases such as HTN and dyslipidemia (16). Therefore, this close association has not been fully uncovered and it needs further research to establish a better understanding of this relationship. Moreover, Most of those studies have been conducted in different populations but less we know about studies conducted on the CKD population which encourages conducting research among this type of population.

CKD is not simply a renal condition; it is a multifaceted systemic metabolic disorder marked by specific changes in lipid and SUA homeostasis that significantly differ from the patterns seen in the general population. These metabolic disturbances unique to CKD are of clinical importance, as they independently accelerate the decline of renal function and increase cardiovascular morbidity, which is the primary leading cause of death in this population. The components of metabolic syndrome (MetS) are notably prevalent among CKD patients, impacting around one-third of this population (17), and their existence is a strong predictor of progression to end-stage kidney disease (ESKD). In particular, MetS is associated with a 2.31-fold increased risk of CKD progression when compared to individuals without MetS (18), with Cox regression analyses validating it as an independent risk factor for the initiation of renal replacement therapy, in conjunction with proteinuria and hyperphosphatemia (17).

In this metabolic context, SUA stands out as a critical yet frequently underestimated factor. Recent large-scale cohort investigations establish SUA as the foremost predictor of incident CKD among the components of MetS (adjusted HR=1.85), outpacing the risks associated with HTN (HR=1.69) or hyperglycemia (HR=1.65) (18). This correlation is mechanistically sound, as HUA incites renal inflammation, endothelial dysfunction, and oxidative stress within the renal system, thereby directly facilitating tubulointerstitial fibrosis and glomerulosclerosis. Additionally, the metabolic relationship between SUA and lipids in CKD reveals unique traits; non-targeted metabolomics uncovers significant disruptions in glycerophospholipid metabolism pathways in CKD patients with simultaneous HUA, differentiating them from either condition alone and indicating a synergistic nephrotoxic impact. Crucially, these associations display gender-specific characteristics; the elevation of SUA correlates with negative cardiac remodeling in female CKD patients, but this is not the case for males (19). Meanwhile, HDL-c’s protective effects on the kidneys show a continuous linear relationship in women, contrasting with a threshold effect in men (20). Given that standard lipid-lowering treatments often provide insufficient cardiovascular protection in CKD, understanding the SUA-lipid relationship could identify innovative therapeutic targets to alleviate metabolic toxicity and disrupt the harmful cycle of renal deterioration. Thus, examining the gender-dimorphic links between SUA and atherogenic lipid fractions (LDL-c, TG) alongside protective HDL-c in CKD is not only physiologically compelling but also crucial for formulating precision-medicine strategies for this vulnerable population.

These findings suggest that elevated SUA levels may contribute to dyslipidemia in CKD patients. Mechanistically, these changes may occur via various pathways in UA-dyslipidemia interaction. The activation of insulin resistance as a possible mediator of SUA could lead to increased TG synthesis and decreased lipoprotein lipase activity, with the potential to block cholesterol metabolism in the liver accompanying TC elevation. In addition, the activation of inflammatory pathways whereby SUA affects lipid metabolism is variously defended, thus aggravating lipid metabolism pathologies in CKD. Herein lies the importance of the relationship between SUA and lipid profiles in CKD patients toward the effective management of dyslipidemia in this population. Interventional measures addressing lifestyle changes through dietary modification, and weight loss, together with drug therapy to lower SUA in HUA patients may improve lipid pattern status and decrease the risk for cardiovascular problems in patients suffering from CKD.

Understanding the relationship between SUA and lipid profiles in CKD is crucial for uncovering potential metabolic interactions and informing clinical managements. To address this gap, we conducted our research to explore this relationship between SUA levels and lipid profiles in the CKD population.

## Methods

### Study design and participant eligibility

We conducted a multi-center, prospective observational study, involving 374 non-dialysis stage 1/4 CKD patients from December 2023 to March 2025. The study was carried out in three tertiary hospitals located in Xuzhou, China: Xuzhou Medical University Affiliated Hospital, the Second Affiliated Hospital of Xuzhou Medical University, and Fengxian Peoples Hospital. Standardization was achieved through: 1- Centralized training for site investigators on uniform protocols; 2- Stringent quality control with external validation; 3- A steering committee overseeing biweekly patient enrollment and endpoint adjudication. Centralized eligibility verification guaranteed consistent CKD staging. This design utilized diverse catchment areas while reducing site-specific bias; we performed comparisons of baseline characteristics and outcomes across the participating centers and found no significant differences in lipid profiles and SUA. Throughout sensitivity analyses, which included the three centers, we consistently found that our results were similar. Eligible patients were divided into male group n=210 and female group n=164. Patients were healthy, and no severe CVD was observed. CKD was diagnosed per KDIGO criteria (21) with confirmed eGFR <60 mL/min/1.73m² for ≥3 months. Any patient with HUA which defined as SUA >420 μmol/L, according to the Guidelines for the Diagnosis and Management of HUA in China, were excluded (5) to isolate SUA-lipid relationships in non-HUA populations, recognizing this may limit generalizability. Inclusion criteria were as follows [1] Xuzhou residency ≥12 months; [2] Age 20–80 years; [3] CKD stages 1/4; [4] No urate-lowering/statin therapy for ≥3 months; [5] No severe cardiac history. Exclusion criteria were [1] patients undergoing dialysis therapy, due dialysis therapy can alter the level of SUA and lipid profiles, [2] pregnancy and lactation period, [3] patients taking drugs that affect SUA concentration and lipid metabolism, [4] patients with severe cardiovascular and neurological complications, [5] patients with gout and those undergoing anti-gout treatment and immunosuppressive therapy, [6] severe liver disease, [7] patients with missing data on SUA and lipid profiles and those who are not fit to participate in the study, [8] diabetic patients were excluded, no cases were enrolled due to high comorbidity burdens affecting eligibility. Patients were followed for one month to observe if they are suitable for enrolment. Participants in the study were receiving their conventional therapy for CKD and no interference with their medication administration. The study protocol was approved by the scientific research ethics committee of the Faculty of Nephrology, Affiliated Hospital of Xuzhou Medical University project ethics number (XYFY2024-KL642-01) and was registered on the Chinese Clinical Trial (ChiCTR2500096252), and the study was conducted in accordance with the Declaration of Helsinki. All participants enrolled in the study gave written informed consent and all experiments were performed in accordance with relevant guidelines and regulations.

### Sample size calculation

GPower was utilized to calculate the sample size so that a precise estimation of the outcomes could be made and the associations could be detected reliably. For prevalence estimation, assuming a 95% confidence level (Z =1.96), maximum variability (*P=0.5*), and a 5% margin of error, yielded 385 participants. The enrollment of 374 patients provided a 5.1% margin of error, which can be accepted for cross-sectional studies. For analytical purposes, this sample size provides ≥80% power (α=0.05) to detect clinically meaningful odds ratios ≥2.0 in logistic regression models, assuming a baseline outcome prevalence of 20% and ≤10 predictors. The final sample also accounts for possible missing data and balances feasibility against robust measurement precision and hypothesis-testing power (22).

### Diagnostic criteria

Participants’ CKD stages were determined according to their eGFR which was calculated according to the Chronic Kidney Disease Epidemiology Collaboration formula CKD-EPI which is based on serum creatinine levels, gender, and age at the time of enrolment (23). HUA was defined as SUA more than 420 μmol/L in men and 360 μmol/L in females which is a wildly accepted criterion for HUA (24, 25). Patients were classified as diabetic if they had fasting blood glucose (FBG) ≥ 7.0 mmol/L or higher (26). Participants with systolic blood pressure (SBP) ≥ 140 mmHg and diastolic blood pressure (DBP) ≥ 90 mmHg were diagnosed with HTN (27).

### Data collection

The patients’ basic information, demographic data, and medical records were collected by our department medical staff at the time of the visit. Clinical variables include age, weight, BMI, height, past and current medical history, medication administration, etiology of CKD, and CVD such as (HTN [defined as BP ≥140/90 mmHg or antihypertensive use], atherosclerosis, heart failure [clinical diagnosis + echocardiographic confirmation], and myocardial infarction) and other diseases, were also collected and confirmed by the medical staff of our departments. Venous blood samples were obtained after a fasting period of 12 hours and were subsequently analyzed within 24 hours using an automated biochemical analyzer (Roche cobas8000). The laboratory variables included: (SUA, μmol/L; measured through enzymatic colorimetry), (eGFR, ml/min/1.73m²; calculated via the CKD-EPI equation), blood urea nitrogen (BUN, mmol/L), serum creatinine (Scr, μmol/L), and lipid profiles that encompassed total cholesterol (TC, mmol/L; determined by the CHOD-PAP method), triglycerides (TG, mmol/L; evaluated using the GPO-PAP method), high-density lipoprotein cholesterol (HDL-c, mmol/L; assessed through a direct enzymatic assay), and low-density lipoprotein cholesterol (LDL-c, mmol/L; calculated with the Friedewald formula when TG <4.5 mmol/L, otherwise measured directly). Echocardiographs were also obtained from participants and were performed by our hospitals specialists, who are experienced technicians using standardized methods to ensure patients has no severe cardiovascular conditions, tests included left ventricular ejection fraction (LVEF), left ventricular end-diastolic dimension (LVEDD), and left ventricular posterior wall (LVPW) to confirm the absence of severe cardiovascular lesions (defined as LVEF<30%, severe valvular disease, or ventricular aneurysms). The variables were collected and measured according to the American Society of Echocardiography guidelines (28, 29).

### Statistical processing

All data were processed using the SPSS 26.0 statistical software package. Normally distributed data were presented as mean ± standard deviation, while count data were analyzed using a chi-square test, and skewed variables presented as median [IQR] and analyzed with Mann-Whitney U test/Kruskal-Wallis test. Continuous variables were assessed for normal distribution using both graphical techniques (Q-Q plots in conjunction with detrended normal plots) and statistical assessments (Kolmogorov-Smirnov test for n>50; Shapiro-Wilk test for n ≤ 50). Normality was confirmed if the Q-Q plots displayed a linear configuration of data points along the diagonal, and the statistical tests produced *P>0.05*. The data were then re-evaluated until the criteria for normality were met. Differences between age groups and SUA quartiles were compared using a one-way analysis (ANOVA) of variance. The correlation between SUA and lipid profiles was analyzed using Spearman’s correlation analysis and linear regression. Multiple regression analysis was utilized to study the risk factors and predictors of SUA. Tukey’s Honestly Significant Difference (HSD) analysis was utilized for comparing group mean differences between quartiles Q1-Q4. Multivariable linear regression models were used to assess SUA-lipid associations, the selection of adjusted variables age, gender, BMI, and lipid profiles followed a predefined dual rationale, by implementing adjustments with interaction to control for confounders, with SUA×gender interaction terms to test effect modification through variance inflation factors (VIFs) were <5, and residual diagnostics. first, clinical relevance inclusion of factors with established biological links to SUA-lipid metabolism in CKD age/gender modify uric acid clearance; BMI influence dyslipidemia pathogenesis; and statistical protocol which includes retention of covariates altering SUA-lipid β-coefficients by ≥10% in univariate screening. Missing data were effectively reduced by strict adherence to protocols: there were no missing SUA or lipid profile measurements due to our exclusion criteria that mandated complete laboratory data. For baseline covariates, we utilized complete-case analysis after verifying that the data were missing completely at random (Little’s MCAR test: χ²=3.21, *P=0.78*). No imputation was conducted due to the extremely low level of missingness (<0.5% of covariates) and the clinical insignificance of imputing minimally missing demographic information. P values less or equal to <*0.05* indicates a statistically significant difference. GraphPad Prism 9 was used to generate graphs.

## Results

### Characteristics of participants stratified according to gender

**Figure 1:**
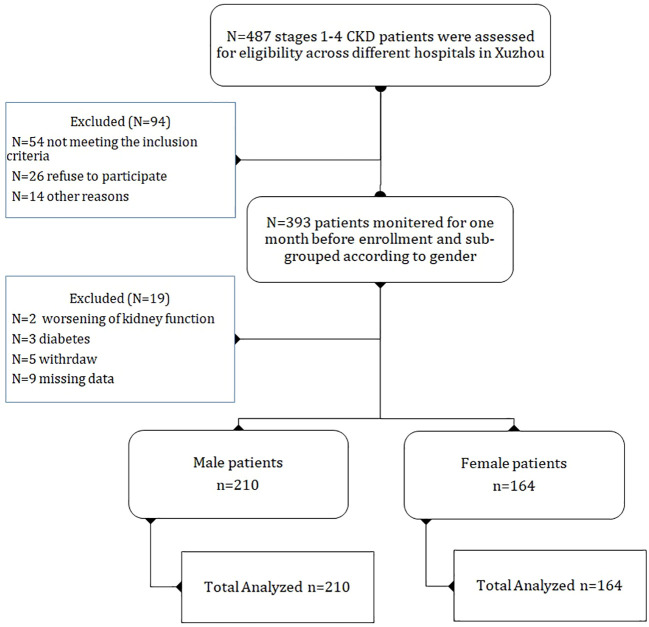
The flow chart of the current study outlines the evaluation process for 487 stage 1/4 chronic kidney disease patients. Patients were evaluated and assessed for eligibility, patients who met the inclusion criteria were monitored for one month before enrollment. After enrollment, further sub-groups were formed based on gender. Patients with any kind of missing data were excluded from the study.

Refer to (**Figure 1**) for the flow chart of the current study. There were 374 participants in the current study, consisting of 210 (56%) males with SUA level of (321.71 ± 34.77μmol/L) and mean age (53.49 ± 13.57 years), and 164 (43%) females with SUA level of (265.38 ± 29.50μmol/L) and mean age (53.18 ± 13.66 years) and the difference was statistically significant between both groups (*P<0.001*). The male group had a BMI of (26.50 ± 2.48 kg/m²) and the female group had (25.28 ± 1.80 kg/m²) with a statistically significant (*P<0.001*). The CKD etiology in this population was primarily chronic glomerular nephritis, 135 (36%) in the male group and 102 (27%) in the female group, and HTN nephropathy was found in 58 (15%) in the male group and 42 (11%) in the females group. CKD stages 1–2 was found in 13 (5%) male patients and 7 (2%) female patients with statistically difference (*P<0.001*). The majority of participants were stage CKD stage 3/4, with 197(52%) male patients and 157 (41%) female patients with statistically significant (*P<0.001*). For more baseline characteristics, please refer to (**Table 1**).

**Table 1:** Baseline characteristics in the study population stratified according to gender.

Characteristics	All participants	Male participants	Female participants	U/t/χ²	P value
Patients No.	N=374	N=210	N=164		
Demographical					
Age Years	53.35 ± 13.69	53.49 ± 13.75	53.18 ± 13.66	0.21	0.832
Body weight kg	71.16 ± 9.80	78.70 ± 5.58	61.51 ± 3.60	34.24	0.001
Height cm	165.26 ± 9.11	172.46 ± 4.71	156.04 ± 3.04	38.72	0.001
BMI kg/m²	25.97 ± 2.29	26.50 ± 2.48	25.28 ± 1.80	5.29	0.001
Etiology of CKD					
Hypertension Nephropathy (%)	100 (27)	58 (15)	42 (11)	0.19	0.663
Diabetic Nephropathy (%)	0 (0)	0 (0)	0 (0)	N/A	N/A
Chronic Glomerular Nephritis (%)	237 (63)	135 (36)	102 (27)	0.19	0.662
Other (%)	37 (10)	17 (4)	20 (6)	1.74	0.187
Lab-Markers					
SUA μmol/L	297.01 ± 42.91	321.71 ± 34.77	265.38 ± 29.50	16.59	0.001
TG mmol/L	1.62 ± 0.25	1.72 ± 0.22	1.50 ± 0.22	9.13	0.001
TC mmol/L	4.16 ± 0.78	4.48 ± 0.68	3.74 ± 0.70	10.29	0.001
LDL-c mmol/L	2.23 ± 0.46	2.39 ± 0.42	2.02 ± 0.43	8.31	0.001
HDL-c mmol/L	1.41 ± 0.36	1.27 ± 0.32	1.59 ± 0.32	-9.22	0.001
FBG mmol/L	4.98 ± 0.70	4.99 ± 0.70	4.96 ± 0.71	0.44	0.659
Hgb g/L	122.80 ± 12.77	124.35 ± 13.44	120.83 ± 11.61	2.66	0.008
ÆeGFR mL/min/1.73m²	40.51 ± 10.18	40.97 ± 10.43	39.93 ± 9.85	0.98	0.325
Scr μmol/L	167.23 ± 46.81	174.60 ± 46.77	157.79 ± 45.28	3.49	0.001
BUN mmol/L	8.36 ± 2.07	8.85 ± 1.88	7.72 ± 2.14	5.39	0.001
¹C-cys mg/L	1.48[1.32, 1.65]	1.52[1.35, 1.68]	1.45[1.30, 1.62]	1582	0.082
¹ Hs-CRP mg/L	2.80[1.60, 4.20]	2.70[1.55, 4.15]	2.90[1.70, 4.40]	1523	0.401
¹TnT ng/L	5.20[3.80, 7.10]	5.10[3.70, 6.90]	5.30[3.90, 7.30]	1600	0.210
¹ CK u/L	56.0[54.0, 68.0]	62.0[56.0, 70.0]	58.0[52.0, 65.0]	1400	0.001
¹CK-MB ng/mL	1.40[1.20, 1.70]	1.42[1.22, 1.72]	1.38[1.18, 1.68]	1700	0.150
^a^LVEF %	56.95 ± 3.45	56.99 ± 3.45	56.90 ± 3.47	0.26	0.794
^a^LVEDD mm	56.88 ± 3.60	56.99 ± 3.65	56.74 ± 3.53	0.65	0.512
^a^LVPW mm	9.79 ± 1.30	9.90 ± 1.31	9.65 ± 1.26	1.84	0.066
Systolic BP mmHg	127.52 ± 7.25	126.85 ± 8.14	128.37 ± 5.84	-2.02	0.044
Diastolic BP mmHg	85.32 ± 5.96	84.38 ± 5.75	86.52 ± 6.03	-3.48	0.001
Medications					
Antiplatelet agent (%)	121 (32)	64 (17)	57 (15)	0.75	0.386
Diuretics (%)	91 (24)	39 (18)	52 (31)	7.51	0.006
ACEI/ARB (%)	250 (66)	135 (36)	115 (30)	1.39	0.238
β-blocker (%)	77 (20)	48 (12)	29 (8)	1.51	0.217
CCB (%)	64 (17)	39 (10)	25 (7)	0.72	0.394
Insulin (%)	0 (0)	0 (0)	0 (0)	N/A	N/A
*Lipid lowering drugs (%)	0 (0)	0 (0)	0 (0)	N/A	N/A
Urate lowering drugs (%)	0 (0)	0 (0)	0 (0)	N/A	N/A
Coexisting conditions					
CKD stage 1/2 (%)	20 (5)	13 (3)	7 (2)	0.68	0.411
CKD stage 3/4 (%)	354 (94)	197 (52)	157 (41)	0.61	0.434
Smoking (%)	143 (38)	84 (22)	59 (15)	0.65	0.421
Alcohol use (%)	182 (48)	126 (33)	56 (14)	10.82	0.001

Measurement data are given as mean ± SD or number (%), ¹Skewed variables presented as median [IQR] and analyzed with Mann-Whitney U test.*P* < 0.05 was deemed statistically significant.

T-test was utilized for continues variables, and Chi-square test (χ²)was utilized for categorical variables.

Æ eGFR (mL/min/1.73m²) was calculated with the according to the Chronic Kidney Disease Epidemiology Collaboration formula CKD-EPI.

a Variables were measured according to the American Society of Echocardiography guideline.

*Lipid lowering drugs Including (satins, ezetimibe, PCSK9).

BMI, body mass index; SUA, serum uric acid; TG, triglyceride; TC, total cholesterol; LDL-c, low density lipoprotein cholesterol; HDL-c, high density lipoprotein cholesterol; FBG, fasting blood glucose; Hgb, hemoglobin; eGFR, estimated glomerular filtration rate; Scr, serum creatinine; BUN, blood urea nitrogen; C-cys, Cystin C; Hs-CRP, High-sensitivity C-reactive protein; TnT, troponin T; CK, creatine kinase; CK-MB, creatine kinase MB; LVEF, left ventricular ejection fraction; LVEDD, left ventricular end-diastolic dimension; LVPW, left ventricular posterior wall; ACEI/ARB, angiotensinogen converting enzyme inhibitor/angiotensin receptor blocker; CCB, calcium channel blocker; CKD, chronic kidney disease; N/A, Not applicable.

### Characteristics of participants stratified according to SUA quartiles

To investigate the association of SUA with lipid profiles, we stratified participants into quartiles based on SUA levels. The quartiles were defined as follows: Q1 (SUA<230μmol/L) including 24 participants (6.41%), Q2 (SUA 231-280μmol/L) included 126 participants (33.68%), Q3 (SUA 281-343μmol/L) included 150 participants (40.10%) and Q4 (SUA 344-400μmol/L) included 74 participants (19.78%). The average age across all quartiles ranged between 47 and 57 years. Interestingly, BMI was highest among Q4 participants compared to other groups with a mean value of (26.53 ± 1.93 kg/m²) which was statistically significant (*P<0.001*). Q1 had the majority of female participants with 22 (5.88%), and only 2 (0.5%) male participants. Q4 was predominantly composed of male participants with 74 (19.78%) and no female participants in this group. SUA levels were higher in men compared to female participants. In contrast, female participants were predominating in the Q2 group 98 (26.20%). Regarding the lipid profiles, TC, TG, and LDL-c significantly increased from Q1 to Q4, with a statistical significance (*P<0.001*). As for the HDL-c, it showed a reverse trend by decreasing from Q1 to Q4 with a statistical significance (*P<0.001*). For more biomarkers and comparisons among the quartile groups, please refer to (**Table 2**).

**Table 2:** Characteristics of patients across different quartiles of serum uric acid.

Characteristics	Q1 SUA<230 μmol/L	Q2 SUA 231-280 μmol/L	Q3 SUA 281-343 μmol/L	Q4 SUA 344-400 μmol/L	P value
Patients No.	N=24	N=126	N=150	N=74	
Demographical					
Age Years	47.21 ± 12.99	52.53 ± 13.68	52.95 ± 14.61	57.57 ± 10.89	0.006
Body weight kg	61.25 ± 5.02	65.02 ± 8.17	73.81 ± 8.96	79.49 ± 4.74	0.001
Height cm	157.67 ± 7.60	159.40 ± 6.96	167.45 ± 8.57	173.27 ± 4.50	0.001
BMI kg/m²	24.66 ± 1.58	25.53 ± 2.21	26.26 ± 2.48	26.53 ± 1.93	0.001
°Male	2 (0.5%)	31 (8.28%)	110 (29.41%)	74 (19.78%)	<0.001
°Female	22 (5.88%)	98 (26.20%)	40 (10.69%)	0 (0%)	<0.001
Lab-Markers					
SUA μmol/L	226.96 ± 6.82	258.78 ± 15.25	310.45 ± 17.12	357.59 ± 13.02	<0.001
TG mmol/L	1.29 ± 0.24	1.53 ± 0.20	1.68 ± 0.24	1.78 ± 0.17	<0.001
TC mmol/L	3.49 ± 0.64	3.81 ± 0.69	4.27 ± 0.81	4.73 ± 0.33	<0.001
LDL-c mmol/L	1.72 ± 0.40	2.02 ± 0.44	2.31 ± 0.42	2.57 ± 0.25	<0.001
HDL-c mmol/L	1.89 ± 0.34	1.53 ± 0.31	1.37 ± 0.32	1.13 ± 0.22	<0.001
FBG mmol/L	4.85 ± 0.85	4.91 ± 0.72	5.00 ± 0.74	5.07 ± 0.70	0.351
Hgb g/L	119.00 ± 11.3	122.00 ± 11.9	122.39 ± 14.4	126.26 ± 10.3	0.042
*eGFR mL/min/1.73m²	43.57 ± 3.16	39.64 ± 9.50	41.61 ± 11.43	38.78 ± 8.48	0.073
Scr μmol/L	133.75 ± 39.46	157.68 ± 44.14	172.19 ± 50.33	184.30 ± 36.42	0.001
BUN mmol/L	6.95 ± 2.03	7.84 ± 2.09	8.58 ± 2.18	9.24 ± 1.24	0.001
¹C-cys mg/L	1.20[1.10, 1.35]	1.45[1.30, 1.65]	1.52[1.35, 1.70]	1.58[1.45, 1.70]	<0.001
¹Hs-CRP mg/L	3.80[2.10, 5.20]	3.20[1.80, 4.60]	3.10[1.90, 4.50]	2.90[1.50, 4.30]	0.495
¹TnT ng/L	5.80[4.20, 7.20]	5.60[4.00, 7.10]	6.20[4.50, 8.00]	5.00[3.50, 6.50]	0.070
¹CK u/L	56.0[50.0, 62.0]	59.0[53.0, 65.0]	63.0[55.0, 70.0]	60.0[54.0, 66.0]	0.079
¹CK-MB ng/mL	1.40[1.20, 1.60]	1.45[1.25, 1.70]	1.42[1.20, 1.65]	1.55[1.30, 1.75]	0.306
^a^LVEF %	58.17 ± 2.58	56.79 ± 3.45	57.63 ± 3.55	55.45 ± 3.00	0.001
^a^LVEDD mm	55.99 ± 3.64	57.33 ± 3.34	55.99 ± 3.85	58.05 ± 3.03	0.068
^a^LVPW mm	9.63 ± 1.27	9.61 ± 1.22	9.95 ± 1.34	9.81 ± 1.33	0.175
Systolic BP mmHg	125.42 ± 4.71	127.95 ± 6.36	128.53 ± 8.00	127.52 ± 7.25	0.008
Diastolic BP mmHg	84.75 ± 5.01	86.17 ± 6.24	85.87 ± 6.07	82.93 ± 4.91	0.001

Measurement data are given as mean± SD, ¹Skewed variables presented as median [IQR] and analyzed with Kruskal-Wallis test. P<0.05 was deemed statistically significant. Q1 serum uric acid [SUA] <230 μmol/L, Q2 SUA 231-280 μmol/L, Q3 SUA 281-343 μmol/L, Q4 SUA 344-400 μmol/L.

One-way analysis (ANOVA) was utilized to compare the variables between all groups.

°gender is presented as percentage (%).

a Variables were measured according to the American Society of Echocardiography guideline.

*eGFR (mL/min/1.73m²) was calculated according to the Chronic Kidney Disease Epidemiology Collaboration formula CKD-EPI.

BMI, body mass index; SUA, serum uric acid; TG, triglyceride; TC, total cholesterol; LDL-c low-density lipoprotein cholesterol; HDL-c, high-density lipoprotein cholesterol; FBG, fasting blood glucose; Hgb, hemoglobin; eGFR, estimated glomerular filtration rate; Scr, serum creatinine; BUN, blood urea nitrogen; C-cys, Cystin C; Hs-CRP, High-sensitivity C-reactive protein; TnT, troponin T; CK, creatine kinase; CK-MB, creatine kinase MB; LVEF, left ventricular ejection fraction; LVEDD, left ventricular end-diastolic dimension; LVPW, left ventricular posterior wall.

### Pair-wise comparisons of dependent variables across four quartiles

**Figure 2:**
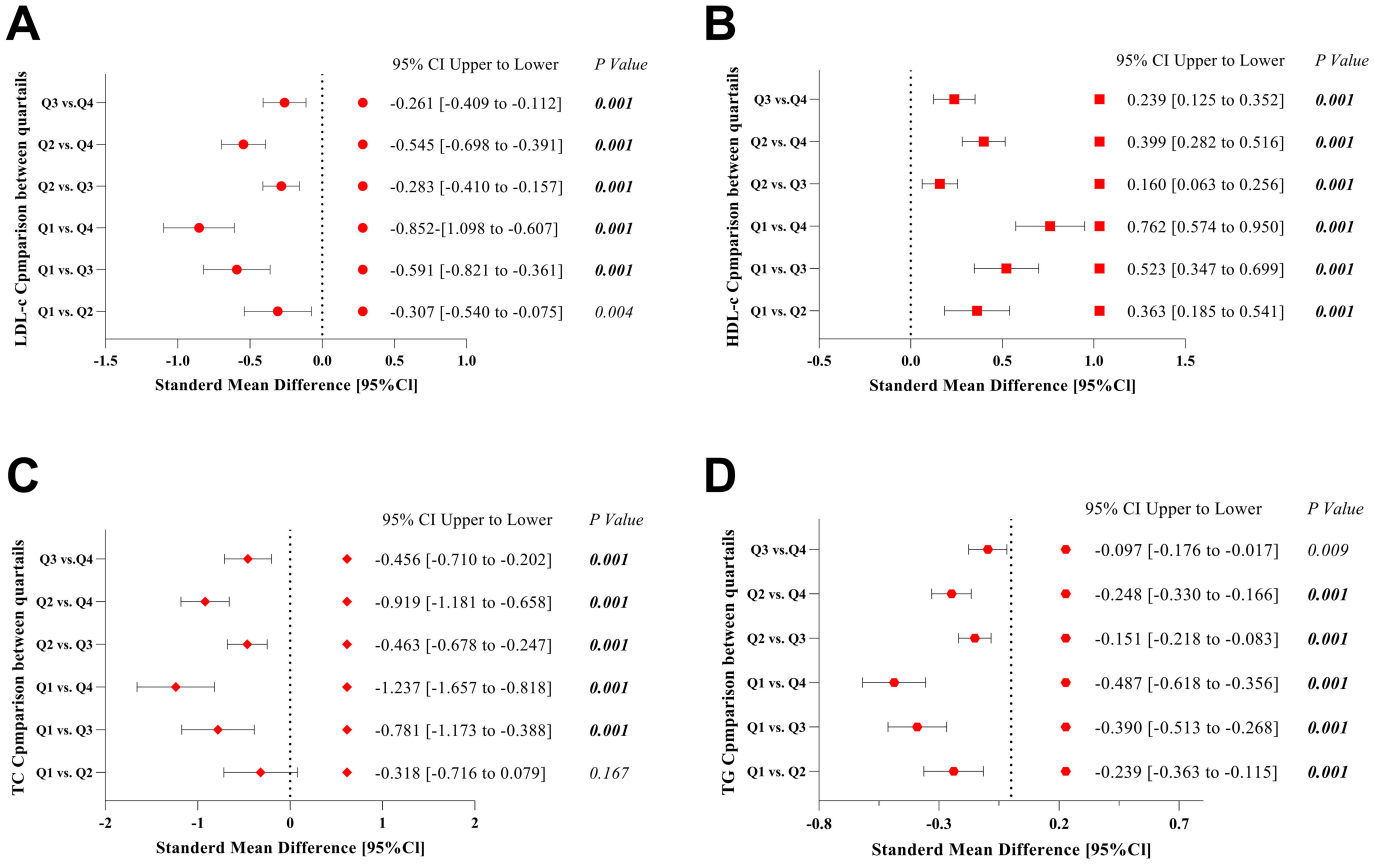
Forest plots of lipid profiles mean differences among the quartiles Q1-Q4. **(A)** Mean differences of LDL-c **(B)** Mean differences of HDL-c **(C)** Mean differences of TC **(D)** Mean differences of TG. Q1 serum uric acid [SUA] <230 µmol/L, Q2 SUA 231-280 µmol/L, Q3 SUA 281-343 µmol/L, Q4 SUA 344-400 µmol/L. TG, triglyceride; TC, total cholesterol; LDL-c, low-density lipoprotein cholesterol; HDL-c, high-density lipoprotein cholesterol, 95%CI, confidence intervals.

The analysis of LDL-c levels of Q1 vs. Q2, Q3, Q4 were significantly lower in Q1 compared to higher quartiles all (*P<0.05*). The largest difference was observed between Q1 and Q4 with mean difference (-0.852, 95% CI [-1.098 to -0.607], *P<0.001*). Q2 vs. Q3, Q4: LDL-c increased progressively across quartiles, with Q4 showing the highest levels, Q2 vs. Q4: mean difference (-0.545, 95% CI [-0.698 to -0.391], *P<0.001*). Q3 vs. Q4 difference was smaller but still significant difference was found, mean difference (-0.261, 95% CI [-0.409 to -0.112], *P< 0.001*) (**Figure 2A**). These results suggest a graded increase in LDL-c across quartiles, with the highest levels in Q4. This may indicate that individuals in higher quartiles have a greater cardiovascular risk due to elevated LDL-c.

The analysis of HDL-c levels of Q1 vs. Q2, Q3, Q4 was significantly lower in Q1 compared to all other quartiles all (*P< 0.001*), with the largest difference between Q1 and Q4 mean difference (0.762, 95% CI [0.574 to 0.950], *P<0.001*). Q2 vs. Q3, Q4, in HDL-c continued to rise across quartiles, Q2 vs. Q4: mean difference (0.399, 95% CI [0.282 to 0.516], *P<0.001*). Q3 vs. Q4: A further increase was observed, mean difference (0.239, 95% CI [0.125 to 0.352], *P<0.001*) (**Figure 2B**). HDL-c levels exhibited a progressive increase from Q1 to Q4, suggesting that higher quartiles may have better cardioprotective lipid profiles.

The analysis of TC levels of Q1 vs. Q2 were not significant (*P= 0.167*). Q1 vs. Q3, Q4: TC was significantly lower in Q1 compared to Q3 with mean difference (-0.781, 95% CI [-1.173 to -0.388], *P<0.001*) and Q4 mean difference (-1.237, 95% CI [1.657 to -0.818], *P<0.001*). Q2 vs. Q3, Q4: TC increased significantly in higher quartiles Q2 vs. Q4: mean difference (-0.919, 95% CI [1.181 to -0.658], *P< 0.001*). Q3 vs. Q4: A significant but smaller difference was observed, mean difference (-0.456, 95% CI [-0.710 to -0.202], *P< 0.001*) (**Figure 2C**). TC levels were consistently higher in Q3 and Q4 compared to Q1 and Q2, except for the non-significant difference between Q1 and Q2. This suggests that TC may not discriminate well between the lowest two quartiles but is significantly elevated in higher-risk groups.

The analysis of TG of Q1 vs. Q2, Q3, Q4 were significantly lower in Q1 compared to all other quartiles (all *P<0.05*), with the largest difference between Q1 and Q4, mean difference (-0.487, 95% CI [-0.618 to -0.356], *P<0.001*). Q2 vs. Q3, Q4: TG continued to rise, Q2 vs. Q4 mean difference (-0.248, 95% CI [-0.330 to -0.166], *P< 0.001*). Q3 vs. Q4: A smaller but still significant increase was observed, mean difference (-0.097, 95% CI [-0.176 to -0.017], *P=0.009*) (**Figure 2D**). TG levels showed a clear stepwise increase from Q1 to Q4, reinforcing the association between higher quartiles and adverse lipid metabolism. These findings suggest that individuals in Q3 and Q4 may have an increased cardiovascular risk.

### Association between SUA and lipid profiles

**Figure 3:**
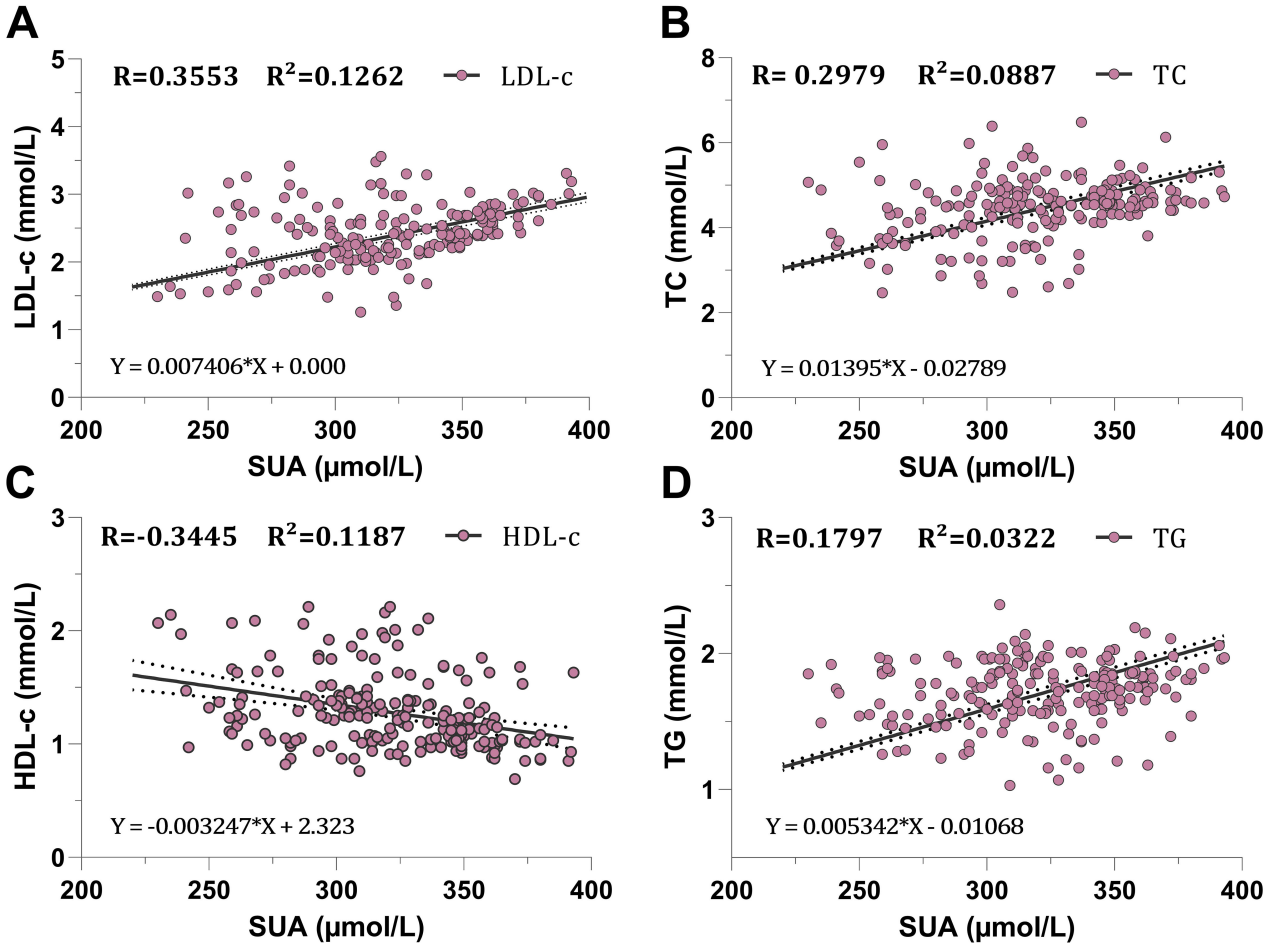
Scatter plot association between serum uric acid and lipid profiles in the male participants. **(A)** correlation with light-density lipoprotein cholesterol, **(B)** correlation with total cholesterol, **(C)** correlation with high-density lipoproteine cholesterol, **(D)** correlation with total triglyceride.

**Figure 4:**
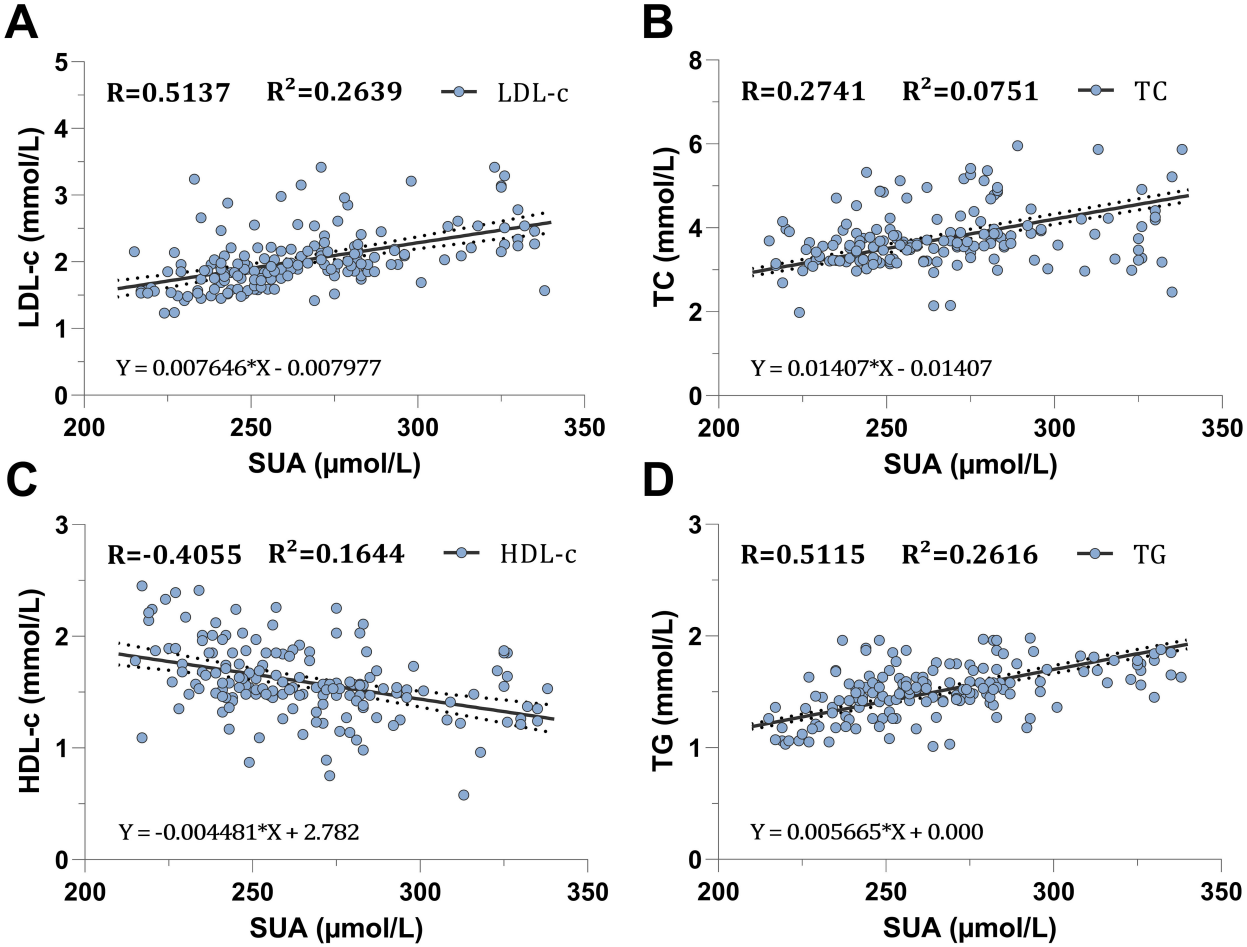
Scatter plot association between serum uric acid and lipid profiles in the male participants. **(A)** correlation with light-density lipoprotein cholesterol, **(B)** correlation with total cholesterol, **(C)** correlation with high-density lipoproteine cholesterol, **(D)** correlation with total triglyceride.

To investigate the correlation between SUA and lipid profiles in greater detail in our CKD population, we conducted a Spearman correlation and simple linear regression analysis within the CKD population. First, we divided the population into male and female groups to examine the association independently. Second, we conducted a comprehensive association analysis for all participants within the population. In the male group, TG, TC, and LDL-c showed a positive correlation with SUA with a statistically significant, correlation coefficient of (R=0.1797, R=0.2979, R=0.3553, respectively; *P<0.0091* for TG and *P<0.0001* for both TC and HDL-c) (**Table 3**, **Figures 3A, B, D**). However, HDL-c showed a negative correlation with SUA with a correlation coefficient of (R=-0.3445, *P<0.0001*) (**Table 3**, **Figure 3C**). Similarly, the female group showed a positive correlation between SUA and TG, TC, and LDL-c with a statistically significant, correlation coefficient of (R=0.5115, R=0.2741, R=0.5137 respectively, *P<0.0004* for TC and *P<0.0001* for both TG and LDL-c) (**Table 3**, **Figures 4A, B, D**). HDL-c also demonstrated a negative correlation with the SUA correlation coefficient of (R=-0.4055, *P<0.0001*) (**Table 3**, **Figure 4C**).

**Table 3:** Association of serum uric acid and lipid profiles based on gender.

Variable	Male Participants N=210			P value	Female Participants N=164			P value
	R	R²	95% CI		R	R²	95% CI	
TG	0.1797	0.0322	0.0453 to 0.3076	0.0091	0.5115	0.2616	0.3887 to 0.6164	<0.0001
TC	0.2979	0.0887	0.1693 to 0.4165	<0.0001	0.2741	0.0751	0.1262 to 0.4101	0.0004
LDL-c	0.3553	0.1262	0.2310 to 0.4682	<0.0001	0.5137	0.2639	0.3912 to 0.6183	<0.0001
HDL-c	-0.3445	0.1187	-0.458 to -0.2193	<0.0001	-0.4055	0.1644	-0.5261 to -0.2690	<0.0001

Simple linear regression analysis was utilized confirm the association between serum uric acid and lipid markers. P<0.05 was deemed statistically significant.

TG, triglyceride; TC, total cholesterol; HDL-c, high-density lipoprotein cholesterol; LDL-c, low-density lipoprotein cholesterol; CI, confidence intervals.

**Figure 5:**
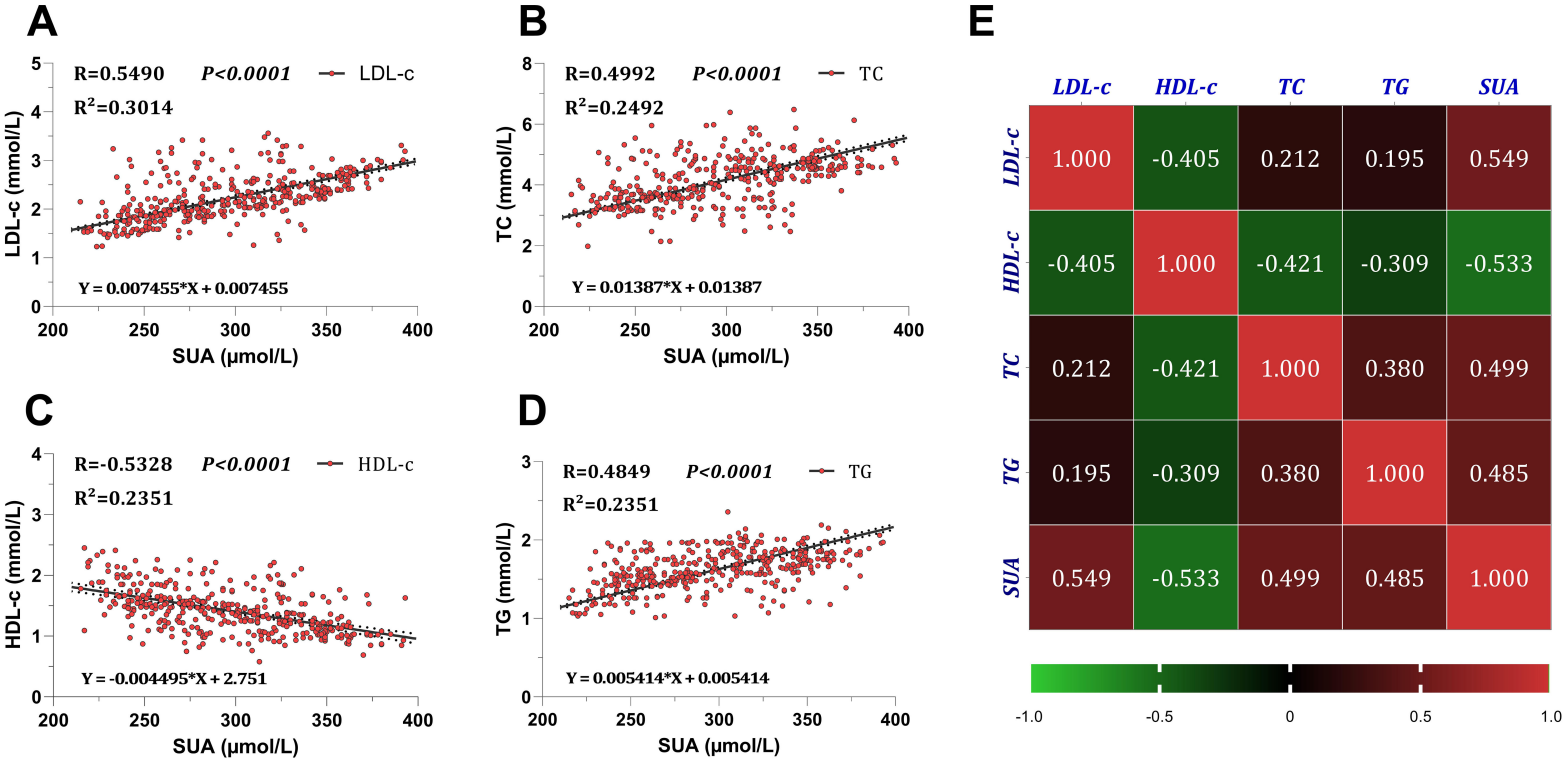
Association between serum uric acid and lipid profiles in the CKD population. **(A)** correlation with LDL-c light-density lipoprotein, **(B)** correlation with TC total cholesterol, **(C)** correlation with HDL-c high-density lipoprotein, **(D)** correlation with TG total triglyceride, **(E)** correlation matrix between SUA and lipids. Simple linear regression and Spearman correlation analyses were used to confirm the association between SUA and lipids. P<0.05 means statistical significance. SUA, serum uric acid; TG, total triglyceride; TC, total cholesterol; LDL-C, low density lipoprotein cholesterol; HDL-c, high density lipoprotein cholesterol.

Comprehensively, Simple linear regression and Spearman correlation analyses revealed a positive correlation between SUA and LDL-c (R=0.5490, [95% CI: 0.4739 to 0.6161], *P<0.001*) (**Figures 5A, E**). Similarly, a positive correlation was found with TC and TG (R=0.4992, [95% CI: 0.4190 to 0.5717], *P<0.001* for TC) (**Figures 5B, E**) and (R=0.4849, [95% CI: 0.4033 to 0.5588], *P<0.001* for TG) (**Figures 5D, E**). While the analyses revealed an inverse correlation between SUA and HDL-c, (R=-0.5328, [95% CI: -0.6017 to -0.4560], *P<0.001*) (**Figures 5C, E**).

The findings demonstrate a strong relationship between SUA and lipid profiles. An increase in the SUA levels may potentially increase LDL-c, TC, and TG while reducing HDL-c in the CKD population.

### Effect size and parameter estimates for lipid profiles across quartiles

We conducted a multivariate analysis to determine the effect of SUA quartiles (Q1-Q4) on multiple dependent variables LDL-c, HDL-c, TC, and TG collectively. The test revealed a significant overall difference in lipid profiles across SUA quartiles. (Pillai’s Trace = 0.548, F=20.60, *P<0.001*), (Wilks’ Lambda = 0.468, F=26.88, *P<0.001*); (Hotelling’s Trace = 1.102, F= 33.58, *P<0.001*), (Roy’s Largest Root = 1.071, F= 98.76, *P<0.001*) (**Table 4**). Roy’s Largest Root, a conservative significance test, demonstrated the strongest effect (Partial η² = 0.517) with 51.7% of the variance in composite lipid outcomes explained by SUA quartiles. The multivariate tests demonstrated strong differences between quartiles (*P<0.001* for all tests) with large to moderate effect sizes (Partial η² = 0.18 0.52). The results are consistent with systemic relationships between SUA levels and multivariate lipid patterns.

**Table 4:** Multivariate analysis of variance for lipid profiles across SUA quartiles Q1-Q4.

Test statistics	Value	F	P value	Partial η²	Effect size
Pillai’s Trace	0.548	20.60	<0.001	0.183	Large
Wilks’ Lambda	0.468	26.88	<0.001	0.223	Large
Hotelling’s Trace	1.102	33.58	<0.001	0.269	Large
Roy’s Largest Root	1.071	98.76	<0.001	0.517	Large

Roy’s Largest Root is an upper-bound F-statistic, providing a lower-bound estimate of significance.

Partial η²: Effect size (0.01 = small, 0.06 = medium, 0.14 = large).

After establishing significant multivariate lipid profile differences among SUA quartiles, *post-hoc* univariate tests were conducted to resolve individual relationships. (**Table 5**) presents adjusted mean differences, confidence intervals, and effect sizes for all lipid variables relative to the highest SUA quartile (Q4, reference group). *Post-hoc* univariate tests revealed significant between-quartile differences for all lipid variables (all *P ≤ 0.01*). Relative to Q4, the lower quartiles of SUA (Q1–Q3) had progressively lower LDL-c, TC, and TG but higher HDL-c levels. The largest between-quartile differences were for LDL-c (Q1 vs. Q4: = -0.853, 95% CI [-1.040, -0.666]) and HDL-c (Q1 vs. Q4: = +0.763, 95% CI [0.620, 0.906]), with both effect sizes being large (Partial η² ≥ 0.18).

**Table 5:** *Post-hoc* univariate analyses of lipid profiles across SUA quartiles Q1-Q4.

Dependent variables	Quartiles	Mean difference (B)	95% CI	P value	Effect size partial η²
LDL-c	Q1	-0.853	-1.040 to -0.666	<0.001	0.18 (Large)
	Q2	-0.545	-0.662 to -0.428	<0.001	0.19 (Large)
	Q3	-0.261	-0.374 to -0.148	<0.001	0.05 (Medium)
	Q4 (Ref.)	0^a^	–	–	–
HDL-c	Q1	0.763	0.620 to 0.906	<0.001	0.22 (Large)
	Q2	0.399	0.310 to 0.488	<0.001	0.17 (Large)
	Q3	0.239	0.153 to 0.326	<0.001	0.07 (Medium)
	Q4 (Ref.)	0^a^	–	–	–
TC	Q1	-1.238	-1.557 to -0.918	<0.001	0.13 (Large)
	Q2	-0.920	-1.119 to -0.720	<0.001	0.18 (Large)
	Q3	-0.457	-0.650 to -0.263	<0.001	0.05 (Medium)
	Q4 (Ref.)	0^a^	–	–	–
TG	Q1	-0.488	-0.588 to -0.388	<0.001	0.19 (Large)
	Q2	-0.248	-0.311 to -0.186	<0.001	0.14 (Large)
	Q3	-0.097	-0.157 to -0.037	0.002	0.02 (Small)
	Q4 (Ref.)	0^a^	–	–	–

Partial η²: Effect size (0.01 = small, 0.06 = medium, 0.14 = large).

MANOVA was conducted first.

0^a^: Q4 reference group (all comparisons were relative to this quartile group).

SUA, serum uric acid; TG, triglyceride; TC, total cholesterol; LDL-c, low-density lipoprotein cholesterol; HDL-c, high-density lipoprotein cholesterol; SE, slandered error; CI confidence interval.

### Characteristics of participants stratified according to age

To investigate the different characteristics of SUA and lipid profiles in the CKD population, we stratified the participants by age groups ranging from 20 to 80 (**Table 6**). Most participants were younger than 60 (35.81% men and 28.33% women). BMI showed an increasing trend with aging, with the lowest value (21.69 ± 1.33kg/m²) observed in the 20–29 age group and the highest value (27.19 ± 2.42kg/m²) seen in the ≥70 age group, which was statistically significant *(P<0.001)*. SUA levels showed an increasing trend with aging in the CKD population, with the lowest value (286.00 ± 36.21 μmol/L) observed in the 20–29 age group, and the highest value (306.04 ± 38.97 μmol/L) was observed in the ≥70 age group with no statistically significant, *(P<0.100*). Interestingly, TG was also higher in the same age group, though this finding was not statistically significant, with a mean value of (1.68 ± 0.22 mmol/L), *(P<0.092)*. LDL-c levels showed similar trending, increased with aging, reaching higher among patients aged ≥70 with a mean value of (2.45 ± 0.51 mmol/L) *(P<0.001)*. HDL-c showed a reverse trend with aging, with the highest value among the young age group 20–29 compared to other age groups with a mean value of (1.57 ± 0.42 mmol/L) and the lowest value (1.37 ± 0.35 mmol/L) was observed in the ≥70 age group with statistically significant, (*P<0.031*). TC showed no significant difference among the age groups, although it was slightly higher among participants aged 60–69 with a mean value of (4.30 ± 0.72 mmol/L) and *(P<0.112)*. eGFR level decreased with aging, being lowest in the ≥70 age group with a mean value of (38.68 ± 11.14 mL/min/1.73m²) and highest value in the 20–29 age group with a mean value of (45.85 ± 11.31 mL/min/1.73m²), which was statistically significant *(P<0.028)*. Body weight increased with aging, being lowest in the 20–29 age group with a mean value of (62.70 ± 6.42 kg) and highest value in the ≥70 age group with a mean value of (73.02 ± 9.99 kg) which was statistically significant *(P<0.001)*. For more details and other biomarkers, please refer to (**Table 6**).

**Table 6:** Characteristics of patients across different age-stratified in the CKD population.

Characteristics	Age-stratified groups (Years)						P value
	20-29	30-39	40-49	50-59	60-69	≥70	
Patients No.	N=23	N=58	N=55	N=104	N=86	N=48	
Demographical							
Body weight kg	62.70 ± 6.42	68.38 ± 10.14	72.13 ± 9.22	72.13 ± 9.58	72.49 ± 9.55	73.02 ± 9.99	0.001
Height cm	169.87 ± 8.65	165.41 ± 9.57	166.24 ± 9.24	164.24 ± 8.77	164.97 ± 9.41	163.69 ± 8.42	0.127
BMI kg/m²	21.69 ± 1.33	24.87 ± 2.01	25.86 ± 1.70	26.52 ± 1.97	26.56 ± 1.65	27.19 ± 2.42	0.001
°Male	16 (4.27)	28 (7.48)	33 (8.82)	57 (15.24)	48 (12.83)	28 (7.48)	0.001
°Female	7 (1.87)	30 (8.02)	22 (5.88)	47 (12.56)	38 (10.16)	20 (5.34)	0.001
Lab-Markers							
SUA μmol/L	286.00 ± 36.21	285.12 ± 38.53	299.05 ± 46.48	297.05 ± 45.71	301.59 ± 42.44	306.04 ± 38.97	0.100
TG mmol/L	1.59 ± 0.24	1.57 ± 0.22	1.68 ± 0.28	1.61 ± 0.26	1.61 ± 0.23	1.68 ± 0.22	0.092
TC mmol/L	3.85 ± 0.78	4.02 ± 0.83	4.18 ± 0.80	4.20 ± 0.76	4.30 ± 0.72	4.10 ± 0.79	0.112
LDL-c mmol/L	2.12 ± 0.50	2.09 ± 0.41	2.19 ± 0.48	2.22 ± 0.45	2.25 ± 0.42	2.45 ± 0.51	¹0.002
HDL-c mmol/L	1.57 ± 0.42	1.50 ± 0.39	1.35 ± 0.35	1.37 ± 0.34	1.42 ± 0.32	1.37 ± 0.35	¹0.031
FBG mmol/L	4.93 ± 0.77	5.01 ± 0.76	5.03 ± 0.62	5.07 ± 0.68	4.80 ± 0.65	5.01 ± 0.81	0.149
Hgb g/L	121.78 ± 14.78	121.60 ± 12.66	125.84 ± 12.36	124.13 ± 11.95	122.79 ± 12.77	118.44 ± 13.35	0.062
*eGFR mL/min/1.73m²	45.85 ± 11.31	41.19 ± 9.52	39.35 ± 9.02	39.18 ± 9.02	42.01 ± 11.25	38.68 ± 11.14	0.028
Scr μmol/L	158.78 ± 50.88	156.29 ± 46.60	167.25 ± 40.35	171.33 ± 45.15	163.24 ± 49.40	182.73 ± 47.95	0.059
BUN mmol/L	7.69 ± 2.77	7.84 ± 2.23	8.39 ± 2.06	8.48 ± 1.82	8.33 ± 2.21	9.04 ± 1.75	0.042
¹C-cys mg/L	1.38[1.25, 1.52]	1.42[1.30, 1.58]	1.50[1.35, 1.65]	1.48[1.33, 1.62]	1.52[1.38, 1.67]	1.56[1.42, 1.72]	0.058
¹Hs-CRP mg/L	3.10[1.80, 4.60]	3.50[2.00, 5.10]	3.00[1.85, 4.40]	2.95[1.80, 4.30]	3.40[2.10, 4.80]	3.00[1.90, 4.20]	0.676
¹TnT ng/L	5.10[3.50, 6.80]	5.60[4.00, 7.20]	5.20[3.90, 6.60]	6.30[4.50, 8.50]	5.80[4.20, 7.50]	5.60[4.00, 7.30]	0.158
¹CK u/L	60.0[54.0, 68.0]	62.0[56.0, 70.0]	58.0[53.0, 64.0]	60.0[55.0, 67.0]	62.0[56.0, 69.0]	57.0[50.0, 64.0]	0.154
¹CK-MB ng/mL	1.38[1.18, 1.60]	1.45[1.25, 1.68]	1.48[1.28, 1.65]	1.42[1.20, 1.63]	1.50[1.30, 1.72]	1.55[1.32, 1.78]	0.398
^a^LVEF %	58.57 ± 3.23	57.05 ± 2.69	56.09 ± 3.34	56.46 ± 3.62	56.90 ± 3.55	58.19 ± 3.54	0.005
^a^LVEDD mm	56.30 ± 3.92	56.78 ± 3.57	57.24 ± 3.38	57.26 ± 3.31	57.00 ± 3.60	55.85 ± 4.22	0.277
^a^LVPW mm	9.61 ± 1.34	9.98 ± 1.29	9.80 ± 1.29	9.76 ± 1.29	9.73 ± 1.28	9.77 ± 1.37	0.857
Systolic BP mmHg	125.35 ± 7.41	127.50 ± 7.52	128.33 ± 7.89	127.16 ± 6.43	127.65 ± 7.18	128.17 ± 7.97	0.640
Diastolic BP mmHg	85.09 ± 5.80	86.40 ± 5.39	85.16 ± 7.18	85.19 ± 5.83	84.87 ± 5.42	85.38 ± 6.52	0.779

Measurement data are given as mean± SD or number (%), ¹Skewed variables presented as median [IQR] and analyzed with Kruskal-Wallis test. *P<0.05* was deemed statistically significant, one way analysis was utilized to compare the variables between all groups.

¹LDL-c and HDL-c showed significant among different age groups.

°gender is presented as percentage (%).

a Variables were measured according to the American Society of Echocardiography guideline.

*eGFR (mL/min/1.73m²) was calculated with the according to the Chronic Kidney Disease Epidemiology Collaboration formula CKD-EPI.

BMI, body mass index; SUA, serum uric acid; TG, triglyceride; TC, total cholesterol; LDL-c, low density lipoprotein cholesterol; HDL-c, high density lipoprotein cholesterol; FBG, fasting blood glucose; Hgb, hemoglobin; eGFR, estimated glomerular filtration rate; Scr, serum creatinine; BUN, blood urea nitrogen; C-cys, Cystin C; Hs-CRP, High-sensitivity C-reactive protein; TnT, troponin T; CK creatine kinase; CK-MB, creatine kinase MB; LVEF left ventricular ejection fraction; LVEDD, left ventricular end-diastolic dimension; LVPW, left ventricular posterior wall.

### Risk factors of SUA in the CKD population

In this section, we seek to investigate the predictors for SUA (dependent variable) in the CKD population on TC, TG, LDL-c, and HDL-c (independent variables). To achieve our results, we conducted multiple regression analyses, which are commonly used to assess the strength of the relationship between dependent and other independent variables. This method will allow us to predict how much variance is being accounted for in a single response of SUA by the set of independent variables. Given that we are examining more than three variables, multiple regressions are the most appropriate analytic approach.

The results of multiple linear regression analysis to predict SUA based on TG showed a coefficient (B=44.689, t=6.806). A significant regression was found, F (4,369) =114.554, *(P<0.001)*. These results direct the positive effects of TG on SUA levels. Moreover, the R² value of 0.261 suggests that for each unit increase in TG, the levels of SUA increase by 26.1% see (**Table 7**). The results of TC were similar, indicating a coefficient (B=12.951, t=5.905), suggesting a positive correlation with SUA levels, with a significant regression found F (4,369) =114.554, *(P<0.001)*. This result highlights a positive correlation between TC and SUA levels, where the R²=0.237 indicates that for each unit increase in TG, the levels of SUA levels increase by 23.7% see (**Table 7**). The regression results to predict SUA levels based on LDL-c were similar, revealing a coefficient of (B=33.454, t=9.562). A significant regression was found, F (4,369) =114.554, *(P<0.001)*, emphasizing a positive relationship between LDL-c and SUA levels. Moreover, the R²=0.365 indicates that for each unit increase in LDL-c, the levels of SUA levels increase by 36.5% see (**Table 7**). Interestingly, the regression results for HDL-c revealed a negative correlation, with a coefficient B (-24.270, t=-4.939). This result indicates that HDL-c is inversely related to SUA levels; moreover, the R²=-205, means for every unit increase in HDL-c, SUA levels decrease by 20.5% see (**Table 7**). These results indicate robust evidence supporting the role of lipid profiles as risk factors for SUA in the CKD population.

**Table 7:** Multiple regression analysis for the risk factors for serum uric acid.

Variables	CKD population N=374			P value	Hypothesis supported
	Coefficient (B)	Stander coefficients (R²)	t-value		
TG mmol/L	44.689	0.261	6.806	0.001	Yes
TC mmol/L	12.951	0.237	5.905	0.001	Yes
LDL-c mmol/L	33.454	0.365	9.562	0.001	Yes
HDL-c mmol/L	-24.270	-0.205	-4.939	0.001	Yes

F=114.554, R Square=0.554.

BMI, body mass index; TG, triglyceride; TC, total cholesterol; LDL-c low density lipoprotein cholesterol; HDL-c, high density lipoprotein cholesterol.

To investigate various risk factors for SUA in both genders, four models were developed. Model one was adjusted for age, the results revealed a significant correlation with (R=0.176, *P<0.010*) for males, and (0.231, *P<0.003*) for females, respectively (**Table 8**). Model two included adjustment for age and BMI, resulting in a significant correlation of (R=0.211, *P<0.009*) in males, and (0.241, *P<0.008*) in females, respectively (**Table 8**). Model three was adjusted for age, BMI, and lipid profiles, the results revealed a significant difference with (R=0.549, *P<0.001* and 0.654, *P<0.001*) in males and females, respectively (**Table 8**). Model four was adjusted for age, BMI, lipid profiles, and weight. The results indicated a significant correlation with (R=0.554, *P<0.001* and R=0.654, *P<0.001*) in males and females, respectively (**Table 8**).

**Table 8:** Multiple regression analysis for the risk factors for serum uric acid based on gender.

Variables	CKD population				P value	Hypothesis supported
	R	R²	Adjusted R²	Std. error		
Male group						
Model 1	0.176	0.031	0.026	34.313	0.010	Yes
Model 2	0.211	0.044	0.035	34.160	0.009	Yes
Model 3	0.549	0.301	0.280	29.498	<0.001	Yes
Model 4	0.554	0.307	0.283	29.443	<0.001	Yes
Female group						
Model 1	0.231	0.053	0.047	28.799	0.003	Yes
Model 2	0.241	0.058	0.246	28.816	0.008	Yes
Model 3	0.654	0.428	0.406	22.744	<0.001	Yes
Model 4	0.654	0.428	0.402	22.813	<0.001	Yes

Models were adjusted differently, male group (n=210), female group (n=164).

Model 1 adjusted for age.

Model 2 adjusted for age and BMI.

Model 3 adjusted for age BMI, LDL-c, HDL-c, TC, and TG.

Model 4 adjusted for age, BMI, LDL-c, HDL-c, TC, TG and weight.

## Discussion

This study reveals a positive correlation between SUA and atherogenic lipid profiles, specifically LDL-c, TC, and TG, in patients with CKD, while showing inverse relationships with HDL-c. Furthermore, a progressive increase in lipid parameters across the quartiles of SUA, particularly for TG, where the levels in the Q4 surpassed established clinical thresholds. These results establish SUA as both a biomarker and a potential mediator of dyslipidemia associated with CKD, carrying significant implications for the stratification of cardiovascular risk. This adds to a growing body of literature suggesting that SUA is crucial to metabolic dysregulation, especially in CKD individuals. This relationship presented with lipid profiles can be explained using varied pathophysiological mechanisms. Elevated levels of SUA could create oxidative stress and inflammation; both could affect the lipid metabolism process (30). SUA could also inhibit endothelial function and diminish lipoprotein lipase activity to generate a build-up of atherogenic lipids in patients with CKD. These effects are further intensified by renal insufficiency regarding the clearance of both SUA and lipids, which thus establishes an exacerbating cycle supporting the development of cardiovascular complications and the progression of CKD. The present study supports several earlier studies that have focused on the relationship of SUA with lipid metabolism in different populations. For instance, in a meta-analysis, Chen et al. (31) documented an association between SUA and dyslipidemia among Chinese adults with basic similarities in the elevation of LDL and TG; however, our study was conducted primarily on the CKD population, and to our knowledge, there are no studies conducted to evaluate this relationship in CKD populations which presents new insight and novelty to the field. Furthermore, Ahlawat M et al. (32) revealed a positive correlation between SUA and LDL-c in the hypertensive population, with a coefficient (R=0.269, *P<0.007*). Our results showed a close correlation between SUA and LDL-c with a coefficient (R=0.3553, *P<0.0001* in men, and R=0.5137, *P<0.0001* in women, respectively) indicating the close correlation between these markers in the CKD population. Similarly, TG and TC levels revealed similar results, indicating a close relationship with SUA. There are some important implications from our results; one is that HDL-c showed a significant inverse correlation with SUA in the CKD population, meaning any increase in the level of HDL-c could potentially decrease SUA levels, other implication is that the positive association between SUA and TG, LDL-c and TC, meaning any decrease in these markers could potentially decrease SUA levels in CKD population and vice versa, and thus could improve both HUA and slow the decline of renal function. Previous findings revealed a pathogenesis among HUA and dyslipidemia, although the populations are different but revealed similar results (14).

This strong correlation between SUA and lipid profiles among the CKD population has extensive clinical implications. This association suggests that SUA can be a potential modifiable risk factor for CVD in this specific population. HUA as an independent risk factor for mortality including CVD (33) and the fact that CVD is the leading cause of mortality in the CKD population, intervention for both SUA and lipid profile levels in the CKD population must be considered, which could have a positive impact on patient’s lives and outcomes among this population. Addressing SUA levels through dietary modifications or ULT could provide benefits in lipid correction, reducing cardiovascular risk and, in return, reducing the progression of CKD (12). Also highlighted by our results is the need for regular follow-up of both SUA and lipids in patients with CKD, particularly with the presence of more severe forms of the disease.

High SUA level, along with its complications such as metabolic syndrome and CVD, presents a major public health challenge globally. The high prevalence of these conditions, accompanied by serious health risks and great economic costs, has raised serious concerns in the public health community (34). Increased levels of SUA are often seen in CKD patients. This increase in SUA levels in CKD patients comes from a combination of renal impairment, metabolic abnormalities, dietary factors, and HTN (35–37). Strategic management of these risk factors through lifestyle changes, dietary modifications, and appropriate pharmacologic treatments, such as ULTs, can significantly mitigate the effects of HUA among CKD patients. Given these facts, SUA levels could act as a biomarker and as a modulator of lipid metabolism (38).

Our research reveals notable gender-specific associations between SUA levels and atherogenic lipid profiles, characterized by positive correlations between SUA and LDL-c/TC in males, and negative correlations between SUA and HDL-c in females. This stratification indicates a mechanistic difference: the more robust SUA-LDL-c relationship in males aligns with findings that HUA promotes hepatic lipogenesis through the activation of xanthine oxidase, while the dissociation of SUA and HDL-c in females may be indicative of estrogen deficiency exacerbating leptin resistance, which in turn hinders HDL-c synthesis. Nevertheless, significant differences arise within populations suffering from diabetic nephropathy. Kosekli MA et al. (39) found that elevated SUA levels were directly correlated with reduced eGFR and increased albuminuria in individuals with type 2 diabetic kidney disease (T2DKD), whereas our investigation excluded diabetic nephropathy due to the complications associated with comorbidities. This distinction is crucial, as the pathophysiology of SUA and lipids in diabetic CKD differs fundamentally from that in non-diabetic CKD.

In another study, Yang et al. (20) demonstrated metabolomic profiling in CKD patients with HUA, confirming these patterns transcend methodological and cohort differences. However, key distinctions arise: we excluded hyperuricemia to isolate non-diabetic pathophysiology, whereas Zhang et al. included both conditions, revealing diabetic cohorts exhibit attenuated SUA-lipid associations due to dominant glucotoxicity. Additionally, Zhou et al. (40) conducted a recent NHANES analysis that identified remnant cholesterol (RC) as a more robust predictor of HUA than conventional lipids (OR = 2.942 for Q4 RC) in the general adult population of the United States. This finding is consistent with our results regarding gender-specific patterns, where males show stronger correlations between SUA and LDL-c/RC, while females display a more significant dissociation between SUA and HDL-c. However, a notable methodological difference between our research and that of Zhou et al. is that we concentrated on non-dialysis CKD stages 1/4 without HUA or diabetic nephropathy, whereas Zhou et al. included individuals with HUA and diabetes, which may elucidate the nonlinear relationship they reported between RC and HUA.

From a methodological perspective, our prospective design enhances cross-sectional evidence, while the exclusion of dialysis patients elucidates the metabolic dynamics prior to ESKD. Nevertheless, subsequent studies ought to confirm these associations within diabetic CKD subgroups and investigate the combination therapy of urate-lowering agents and statins as a potential strategy, which is mechanistically underpinned by the synergies between serum uric acid SUA and lipid pathways.

Our study demonstrated that SUA correlated positively with age among the Chinese CKD population (**Table 8**), likely due to the decline in eGFR levels associated with aging and the presence of CKD. As kidney function declines, this will reduce the ability of the kidneys to excrete SUA and thus lead to accumulation in the blood (30). While some studies showed no correlation between age and SUA (41) this discrepancy could be attributed to the different populations studied, as our research specifically focuses on CKD patients. When adjusted to age and BMI, SUA demonstrated a strong correlation in the CKD population (**Table 8**), indicating that BMI is an independent predictor of SUA levels in CKD patients. This could be explained by higher BMI promoting HUA through mechanisms, including increased SUA production, decreased renal excretion of SUA, and systemic inflammation which can occur through promoting insulin resistance. Rathmann et al. (42) documented that BMI was significantly correlated with increasing SUA in all groups. Although the populations of these studies are different our results showed similar findings. Furthermore, when adjusted to lipid profiles, age, and BMI, SUA showed a significant positive association in Chinese CKD patients (**Table 8**). Several epidemiological studies have shown similar results in different population groups (43, 44). A combination of high LDL-c and low HDL-c worsens SUA levels, highlighting the link between metabolic syndrome and HUA in the context of CKD. With lifestyle modifications, some identified improvements to diet, and pharmacotherapy (45), SUA levels may be regulated to positively influence the health outcomes of CKD patients, controlling SUA in critical for preserving kidney function in non-dialysis CKD population.

## Limitations

While this investigation provides insight and evidence on the correlation of SUA with lipid profiles in the CKD population, certain limitations do need to be stated. First, the study’s observational cross-sectional design inherently restricts the ability to establish causal relationships between SUA levels and lipid profiles, as it only captures associations at a single time point. Second, the study may be subject to selection bias, as the sample population was derived from one city, potentially limiting generalizability to broader CKD populations. Additionally, although we adjusted for key demographic and clinical confounders age, BMI, confounding from unmeasured factors like dietary patterns, genetic polymorphisms in urate transporters, or environmental exposures may persist. Future studies should incorporate comprehensive nutritional assessments, genomic profiling, and physical activity metrics to further refine these relationships. Furthermore, the absence of diabetic nephropathy and HUA patients also limits generalizability, insulin resistance which can significantly influences both SUA and lipid levels were not accounted for and the use of medications such as diuretics, ACE and CCB all which can impact SUA levels and lipid profiles, we deliberately refrained from adjusting for these agents in our statistical models. These drugs are preferentially prescribed to patients with HTN, proteinuria, or advanced CKD conditions intrinsically linked to both SUA elevation and dyslipidemia, statistical adjustment would introduce indication bias, potentially obscuring true biological relationships by correcting for clinical features central to CKD pathophysiology. Consequently, our reported SUA-lipid associations reflect real-world clinical phenotypes.

## Conclusion

The study reveals a significant association between SUA levels and lipid profiles in CKD patients, highlighting a positive correlation with atherogenic lipids LDL-c, TC, and TG and an inverse relationship with cardioprotective lipid HDL-c. SUA quartiles could effectively stratified dyslipidеmia risk, supporting its utility as a biomarker. These findings reveal gender-specific SUA-lipid pathways that may inform personalized cardiovascular risk management in CKD. Notably, lipid profiles were identified as independent predictors for SUA levels in the CKD population. This adds a new dimension to understanding the SUA role in CKD-related dyslipidemia.

## Data Availability

The raw data supporting the conclusions of this article will be made available by the authors, without undue reservation.
